# Supplementary material for: Content-rich biological network constructed by mining PubMed abstracts
Source: BMC Bioinformatics. 2004 Oct 8;5:147. doi: 10.1186/1471-2105-5-147 (PMC528731; doi:10.1186/1471-2105-5-147)
Supplement: Additional File 2 — The original results of the above study (non-essential files are deleted to keep the file size under the limit set by BMC bioinformatics). [file 1471-2105-5-147-S2.bz2 › chilibotAdditionalFile2/dip40/250ID7809159E1008/html/CKS1_CDC2.html]

 


 **CKS1** and **CDC2** 
  
Found 40 abstracts in PubMed, retrieved 40.  
 

 What does Google say? 
 PDF only 
| .edu only 

---

**Interactive relationship** (e.g. stimulation, inhibition, etc)

**Neutral relationship**- We have isolated a Leishmania mexicana homologue of the fission yeast suc1 gene using PCR with oligonucleotides designed to conserved regions of  **cdc2**  kinase subunits cks  [ **CKS1** ] .  Ref: 8670159 Biochem J, 1996
- Our findings support recent models that suggest that Cks  [ **CKS1** ]  proteins target active forms of p34  **cdc2**  to substrates.  Ref: 9632748 Mol Cell Biol, 1998
- All proteins of this family have Cdk  [ **CDC2** ]  binding and anion binding sites, but only mammalian  **Cks1**  binds to Skp2 and promotes the association of Skp2 with p27 phosphorylated on Thr 187.  Ref: 12140288 J Biol Chem, 2002
- This activity, which can be independent of Cdk  [ **CDC2** ]  binding, links Cks  [ **CKS1** ]  to positive growth control pathways regulating the G1 S transition and to cancer.  Ref: 11516665 Curr Biol, 2001
- The interaction of Skp2 with the substrate is further strengthened by the association of the Cdk  [ **CDC2** ]  binding site of  **Cks1**  with Cdk2 cyclin E, to which phosphorylated p27 is bound.  Ref: 12140288 J Biol Chem, 2002
- The Cks  [ **CKS1** ]  Suc1 proteins associate with CDK  [ **CDC2** ]  cyclin complexes, but their precise function s is not well defined.  Ref: 11463388 Mol Cell, 2001
- Xe p9, a Xenopus Suc1 Cks  [ **CKS1** ]  protein, is essential for the  **Cdc2**  dependent phosphorylation of the anaphase promoting complex at mitosis.  Ref: 9716407 Genes Dev, 1998
- Here we review previous observations and recent data that suggest CKS  [ **CKS1** ]  proteins serve as adaptor proteins that modify the functions of  **cdc2**  throughout the cell cycle.  Ref: 9552390 Prog Cell Cycle Res, 1996
- iii in vitro, the phosphorylated forms of CDK1  [ **CDC2** ]  and CDK2 were preferentially linked to CKS  [ **CKS1** ] .  Ref: 10360827 Int J Cancer, 1999
- Phosphate and the phosphate analog metavanadate bind CksHs1 in a shallow pocket and interact with five conserved residues Lys11, Arg20, Ser51, Trp54 and Arg71 suggesting a specific Cks  [ **CKS1** ]  recognition site for a phosphorylated Cdk  [ **CDC2** ]  residue.  Ref: 7791211 J Mol Biol, 1995
- The small Suc1 Cks  [ **CKS1** ]  protein is a ubiquitous subunit of Cdk  [ **CDC2** ]  cyclin complexes, but its precise function has remained unclear.  Ref: 8666234 Genes Dev, 1996
- The beta hinge identified in the  **Cks1**  structure includes a novel pivot and exposes a cluster of conserved tyrosine residues that are involved in Cdk  [ **CDC2** ]  binding but are sequestered in the beta interchanged Cks  [ **CKS1** ]  homolog suc1 dimer structure.  Ref: 10997903 Structure Fold Des, 2000
- We have purified, from starfish oocytes, a 15 kDa protein, p15 CDK  [ **CDC2** ]  BP, which cross reacts with anti Cks  [ **CKS1** ]  antibodies L.  Ref: 12007796 Biochim Biophys Acta, 2002
- p13 suc1 Cks  [ **CKS1** ]  proteins have been implicated in the regulation of cyclin dependent kinase CDK  [ **CDC2** ]  activity.  Ref: 10913169 Mol Cell Biol, 2000
- Here we show that the missing factor is CDK  [ **CDC2** ]  subunit 1  **Cks1** , which belongs to the highly conserved Suc1 Cks  [ **CKS1** ]  family of proteins that bind to some CDKs and phosphorylated proteins and are essential for cell cycle progression.  Ref: 11231585 Nat Cell Biol, 2001
- We show here that  **Cks1**  is required for the protein kinase activity of budding yeast G 1 cyclin CDK  [ **CDC2** ]  complexes.  Ref: 10913169 Mol Cell Biol, 2000
- The suc1 Cks  [ **CKS1** ]  proteins are well conserved regulatory components of cyclin dependent kinases 1 and 2 CDK1  [ **CDC2** ]  2.  Ref: 12007796 Biochim Biophys Acta, 2002
- The  **cdc2**  kinase subunit cks  [ **CKS1** ]  proteins play an essential function in the control of mitosis through their molecular complexes with the  **cdc2**  kinase.  Ref: 8611549 Biochemistry, 1996
- At mitosis, mutant p53 proteins interfere with the control of the metaphase to anaphase progression by up regulating the expression of  **CKs1** , a protein that mediates activatory phosphorylation of the anaphase promoting complex APC by  **Cdc2** .  Ref: 10809376 Histol Histopathol, 2000
- All three binding sites of  **Cks1**  are required for p27 ubiquitin ligation and for the association of Skp2 with Cdk  [ **CDC2** ]  bound, Thr 187 phosphorylated p27.  Ref: 12140288 J Biol Chem, 2002
- The functionality of the Arabidopsis Suc1  **Cks1**  homologue, designed Cks1At, was demonstrated by its ability to rescue the temperature sensitive  **cdc2**  L7 strain of fission yeast at low and intermediate expression levels.  Ref: 9276444 FEBS Lett, 1997
- Assembly of protein complexes with cyclin Cdk  [ **CDC2** ]  is noted and the capacity of the cyclin dependent kinase subunit Cks  [ **CKS1** ] , in such complex, to extend the range of Cdk  [ **CDC2** ]  substrates is documented and discussed in terms of cell cycle regulation.  Ref: 11732181 Protoplasma, 2001
- The role of  **Cks1**  in promoting G 1 cyclin CDK  [ **CDC2** ]  protein kinase activity both in vitro and in vivo provides a simple molecular rationale for the essential role of  **CKS1**  in progression through G 1 phase in budding yeast.  Ref: 10913169 Mol Cell Biol, 2000
- Mutational analyses including in vivo rescue of  **CKS1**  disruption support the dual functional roles of the beta hinge residue Glu94, which participates in Cdk  [ **CDC2** ]  binding, and of the anion binding pocket that is located 22 A away and on an opposite face to Glu94.  Ref: 10997903 Structure Fold Des, 2000
- **Cks1** , a subunit of cyclin dependent kinases, has now been identified as an essential cofactor in the ubiquitination of the Cdk  [ **CDC2** ]  inhibitor p27 by the SCF Skp2 ubiquitin ligase.  Ref: 11516665 Curr Biol, 2001
- ... overexpression of cdc28 protein kinase 1  [ **CKS1** ]   **CKS1**  blocks the inhibition of the cyclin D1 CDK4 complex by the CDK  [ **CDC2** ]  inhibitor p27 Kip1.  Ref: 12605034 Diagn Mol Pathol, 2003

**Non-interactive relationship** (e.g. studied together, co-existance, homology, etc.)

- This latter process requires the Skp2 and anion binding sites of  **Cks1** , but not its Cdk  [ **CDC2** ]  binding site.  Ref: 12140288 J Biol Chem, 2002
- Human CDK  [ **CDC2** ]  subunit 1  **Cks1**  and S phase kinase associated protein 2 Skp2 are components of the SCF Skp2 complex, which acts as a ubiquitin ligase for p27 Kip1.  Ref: 12670508 Biochem Biophys Res Commun, 2003
- The  **Cdc2**  associated Suc1  **Cks1**  protein p9 is also essential for entry of egg extracts into mitosis, but the molecular basis of this requirement has been unknown.  Ref: 10601234 J Biol Chem, 1999
- Furthermore, this altered mitotic spindle checkpoint status was associated with the overexpression of  **Cks1** , a  **Cdc2**  adapter protein that promotes cyclin B degradation.  Ref: 11005810 J Biol Chem, 2000
- The crystal structure of a cyclin dependent kinase Cdk  [ **CDC2** ]  in complex with the human Cks  [ **CKS1** ]  homolog CksHs1 single domain fold revealed the importance of conserved hydrophobic residues and charged residues within the beta hinge motif.  Ref: 10997903 Structure Fold Des, 2000
- The cks  [ **CKS1** ]  proteins for  **cdc2**  kinase subunit are essential cell cycle regulators.  Ref: 7729536 FEBS Lett, 1995
- The Cdk  [ **CDC2** ]  associated protein  **Cks1**  functions both in G1 and G2 in Saccharomyces cerevisiae.  Ref: 8491379 Genes Dev, 1993
- Several new observations in yeast and Xenopus egg extracts together with new structural information tends to enhance the hypothesis that CKS  [ **CKS1** ]  proteins function to alter the activity of  **cdc2**  at several important points in the cell cycle.  Ref: 9552390 Prog Cell Cycle Res, 1996
- This first step occurs independently of PP2A or Suc1 Cks  [ **CKS1** ]  dependent Cdc25  **Cdc2**  association.  Ref: 10523510 J Cell Sci, 1999
- We have investigated the role of proline residues in suc1, a member of the cyclin dependent kinase cks  [ **CKS1** ]  family of proteins, whose known function is to bind to and regulate the activity of the major mitotic cdk  [ **CDC2** ] .  Ref: 10926502 J Mol Biol, 2000
- Here we demonstrate that  **Cks1**  directs the ubiquitin mediated proteolysis of the CDK  [ **CDC2** ]  bound substrate p27Kip1 by the protein ubiquitin ligase E3 SCF Skp2.  Ref: 11463388 Mol Cell, 2001
- Despite the high degree of homology between the nine known CDKs, only CDK1  [ **CDC2** ] , CDK2 and, to a lesser extent, CDK3 are able to bind to the suc1 Cks  [ **CKS1** ]  proteins.  Ref: 12007796 Biochim Biophys Acta, 2002
- CKS  [ **CKS1** ]  proteins, for which the original member, p13suc1, was identified as a suppressor of  **cdc2**  alleles in S.  Ref: 9552390 Prog Cell Cycle Res, 1996
- However, the mechanism by which Cks  [ **CKS1** ]  influences the function of cyclin CDK  [ **CDC2** ]  complexes has remained elusive.  Ref: 10913169 Mol Cell Biol, 2000
- To help elucidate the function of Cks  [ **CKS1** ]  proteins, we examined whether their binding to p34  **cdc2**  the mitotic cdk  [ **CDC2** ]  varies during the cell cycle in Xenopus egg extracts.  Ref: 9632748 Mol Cell Biol, 1998
- We generated mice lacking Cks2, one of two mammalian homologs of the yeast Cdk1  [ **CDC2** ]  binding proteins, Suc1 and  **Cks1** , and found them to be viable but sterile in both sexes.  Ref: 12714746 Science, 2003
- BACKGROUND The p13suc1 gene product is a member of the cks  [ **CKS1** ]  cyclin dependent protein kinase  [ **CDC2** ]  subunit protein family and has been implicated in regulation of the cell cycle.  Ref: 10673427 Structure Fold Des, 2000
- **Cks1**  can both stabilize Cln2 Cdc28 complexes and activate intact complexes in vitro, suggesting that it plays multiple roles in the biogenesis of active G 1 cyclin CDK  [ **CDC2** ]  complexes.  Ref: 10913169 Mol Cell Biol, 2000
- A CDK  [ **CDC2** ]  independent function of mammalian  **Cks1**  targeting of SCF Skp2 to the CDK  [ **CDC2** ]  inhibitor p27Kip1.  Ref: 11463388 Mol Cell, 2001
